# Enhancing the Stability and Photothermal Conversion Efficiency of ICG by Pillar[5]arene-Based Host-Guest Interaction

**DOI:** 10.3389/fchem.2021.775436

**Published:** 2021-10-29

**Authors:** Yue Ding, Chenwei Wang, Bing Lu, Yong Yao

**Affiliations:** School of Chemistry and Chemical Engineering, Nantong University, Nantong, China

**Keywords:** indocyanine green, pillar[5]arene, host-guest interaction, cancer therapy, photothermal

## Abstract

Indocyanine green (ICG) is a classical near-infrared (NIR) photothermal reagent that can be employed in clinical medical detection. Under neutral conditions, ICG can adsorb NIR light effectively for photothermal (PTT) and photodynamic (PDT) therapy. However, ICG is easily degraded in weak acid environments, which seriously restricts its application. In this work, a cationic water-soluble pillar[5]arene (WP5) was selected as the stabilizing agent for ICG. Thanks to the host-guest interaction between WP5 and alkyl sulfonate, the stability and the photothermal conversion efficiency of ICG increased remarkably upon addition of WP5 as investigated by UV-vis spectrum and photothermal studies. Furthermore, an *in vitro* study showed higher efficiency of WP5&ICG in killing cancer cells in a shorter treatment time than the free ICG. Hence, it is hopeful that WP5 can be a new type of supramolecular host in enhancing the stability and photothermal conversion efficiency of photosensitizers.

## Introduction

In today’s society, cancer is one of the world’s most concerning health problems to humans, and the main means of cancer treatment are operation, radiotherapy, and chemotherapy (Issels, 2008; Mcguire, 2016; Song et al., 2015). Operative treatment is highly controllable, but it is limited to large-area tumor tissue removal. Radiotherapy and chemotherapy cause many normal cells to die during the treatment because of their indiscriminate attack on human cells, which can cause side effects and relapse. In recent years, photothermotherapy has attracted tremendous attention due to the fact it can induce tumor cell necrosis at specific sites with minimal invasion and human side effects (Liu et al., 2019; Zheng et al., 2021). Near-infrared radiation (NIR) is particularly beneficial for photothermotherapy because near-infrared light penetrates deeply through tissues, and endogenous biomolecules absorb fewer photons and cause less cell damage in this wavelength range (Cen et al., 2020).

Indocyanine green (ICG) is a kind of low-toxicity photothermal reagent, which has a characteristic absorption peak in the near-infrared region, and its maximum emission wavelength is about 800 nm (Sheng et al., 2013). ICG is widely used in medical diagnosis, such as blood volume, liver function, ophthalmologic angiography, etc. ICG can effectively absorb near-infrared light and convert it into singlet oxygen and heat. Combined with the excellent tissue penetrating ability of near-infrared light and little effect on the tissue itself, ICG can be used in photothermal therapy (PTT) and photodynamic therapy (PDT) (Shafirstein et al., 2012). However, ICG is easily aggregated in aqueous solution, which affects its photothermal conversion and singlet oxygen generation efficiency. In addition, ICG also decomposes rapidly under illumination, especially in weak acid environments, which limits the application prospect of ICG in tumor therapy.

Pillar[5]arenes (Ogoshi et al., 2008; Xue et al., 2012; Ogoshi et al., 2019), composed of hydroquinone or its derivatives bridged by–CH_2_– in the *2,5*-positions, are a smart type of macro-cyclic hosts after crown ethers (Yoo et al., 2019; Zhou et al., 2017), cyclodextrins (Lai et al., 2017), calixarenes (Li et al., 2020), and cucurbiturils (Jiang et al., 2020; Chernikova and Berdnikova, 2020). The preparation and modification of pillar[5]arene is convenient and efficient, which make them outstanding affinity hosts for selectively guests (Chao et al., 2020; Duan et al., 2020; Hao et al., 2020; Sheng et al., 2020; Xu et al., 2020; Guo et al., 2020a; Guo et al., 2020b; Cao et al., 2021; Chen et al., 2021; Shin et al., 2021). Considering the convenient synthesis of pillar[n]arenes and their rich host-guest properties, functional materials based on pillar[n]arenes have been widely studied and applied in various fields, such as drug delivery systems (Cai et al., 2021; Yao et al., 2017; Xiao et al., 2019), molecular machines (Han et al., 2020; Dong et al., 2014; Wan et al., 2020), *trans*-membrane channels (Chen et al., 2013; Strilets et al., 2020), and supramolecular polymeric materials (Xiao et al., 2018; Jie et al., 2020; Zhang et al., 2020). For example, Prof. Huang’s group found that anticancer drug tamoxifen could form a stable complex with water-soluble pillar[6]arene, which will enhance the solubility and bioactivity of tamoxifen (Shangguan et al., 2017). Although pillar[n]arene has been widely applied in the biological field (Yao et al., 2016; Song et al., 2021), the application of pillar[n]arene to improve the stability of photosensitizers has not been investigated. Herein, a cationic water-soluble pillar[5]arene (WP5) was selected to form a complex with indocyanine green (ICG). Due to fact that alkyl sulfonate can be entrapped in the cavity of WP5, ICG was stabilized with its photothermal conversion efficiency increased. We hope this host-guest strategy can be applied in other photosensitizers to prove their stability and activity.

## Experiment Section

### Synthesis of Cationic Water-Soluble Pillar[5]arene

**SCHEME 1:**
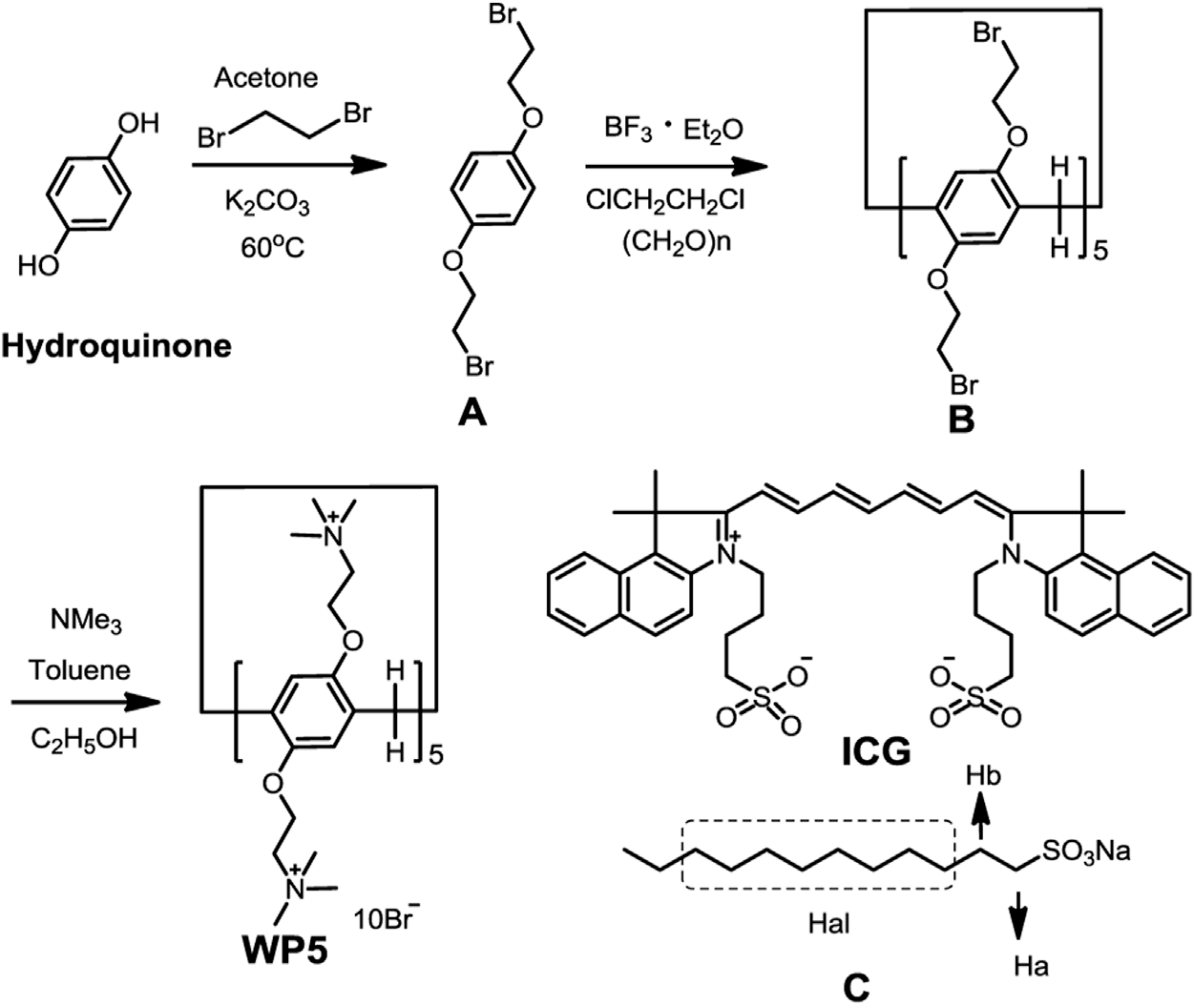
Synthetic route to cationic water-soluble pillar[5]arene and the chemical structures of ICG and model guest C.

As shown in Scheme 1 hydroquinone (5.0 g, 0.045 mol), 1,2-dibromoethane (34.2 g, 0.182 mol), and K_2_CO_3_ (12 g, 0.09 mol) were added in 250 ml of acetone. The mixture was stirred at 60°C for 24 h under an N_2_ atmosphere. When the inorganic solid was removed, the obtained solvent was evaporated and the residue was purified by chromatography on silica gel (petroleum ether/CH_3_COOCH_2_CH_3_, *v*/*v* 50:1) to give **A** as a white crystal. ^1^H NMR (400 MHz, CDCl_3_) as shown in Supplementary Figure S1: δ 6.86 (s, 4H, ArH), 4.25 (t, *J* = 6.3 Hz, 4H, -CH_2_-), 3.62 (t, *J* = 6.3 Hz, 4H, -CH_2_-). Then **A** (5.0 g), paraformaldehyde (3.0 g), and 2.25 ml of BF_3_•Et_2_O were added to 50 ml of ClCH_2_CH_2_Cl and stirred at 25°C until the reaction was finished. Then the saturated NaHCO_3_ solution was added into the mixture. The mixture was separated and the organic phase was collected. The solvent was evaporated and the residue was purified by chromatography on silica gel (petroleum ether/CH_3_COOCH_2_CH_3_, *v*/*v* 25:1) to give **B** as a white solid. ^1^H NMR (400 MHz, CDCl_3_) as shown in Supplementary Figure S2: δ 6.91 (s, 10H, ArH), 4.23 (dd, *J* = 6.3, 5.1 Hz, 20H, -CH_2_-), 3.84 (s, 10H, -CH_2_-), 3.63 (t, *J* = 5.7 Hz, 20H, -CH_2_-). At last, **B** (1.68 g, 1.00 mmol) and trimethylamine (2.36 g, 40.0 mmol) were stirred into 50 ml of dry toluene overnight under reflux. The reaction solvent was evaporated and the residue was recrystallized with CH_3_CH_2_OH (1.72 g, yield: 87.3%). ^1^H NMR (400 MHz, D_2_O), as shown in Supplementary Figure S3, δ 6.92 (s, 10 H, ArH), 4.49–4.32 (m, 20 H), 3.89–3.54 (m, 30 H), 3.33 (m, 90 H, N-CH3). ^13^C NMR (101 MHz, D_2_O) as shown in Supplementary Figure S4, δ: 148.98, 129.59, 116.42, 115.27, 65.49, 64.94, 63.31, 62.71, 54.04, 29.33. MS (m/z): HRMS (ESI) Calcd. for C85H150Br8N10O10 ([M – 2Br]^2+^): 1055.2461, found: 1055.2716 (Supplementary Figure S5). Elemental analysis: N, 6.15%; C, 44.86%; H, 6.59%.

## Materials

All reagents and solvents were commercially available in analytical grade and used as received. Further purification and drying by standard methods were employed and the solvents and reagents were distilled prior to use when necessary. All evaporations of organic solvents were carried out with a rotary evaporator in conjunction with a water aspirator.

## Methods

NMR spectroscopy: ^1^H and ^13^C NMR spectra were recorded with an Aviance III 400 MHz or 600 MHz liquid-state NMR spectrometer.

ESI-MS spectroscopy: Electrospray ionization mass spectra (ESI-MS) were measured by Agilent 6520 Q-TOF-MS.

Fluorescence spectroscopy: Fluorescence spectra were recorded on a Shimadzu HITACHI F-4500 spectrophotometer.

Cell viability: Human cervical cancer cells (HeLa cells) were incubated in Dulbecco’s modified Eagle’s medium (DMEM). The medium was supplemented with 10% fetal bovine serum and 1% penicillin-streptomycin. HeLa cells were seeded in 96-well plates (5 × 10^4^ cell ml^−1^, 0.1 ml per well) for 24 h at 37°C in 5% CO_2_. Then the cells were incubated with WP5⊃ICG for 24 h. The relative cellular viability was determined by the MTT assay.

Confocal laser scanning microscopy: HeLa cells were seeded in 6-well plates (5 × 10^4^ cell ml^−1^, 2 ml per well) for 24 h at 37°C in 5% CO_2_. The cells were incubated with the corresponding solution for 4 h. Then the medium was removed, and the cells were washed with phosphate buffer solution three times. Finally, the cells were subjected to observation by a confocal laser scanning microscope.

Photothermal conversion: For measuring the photothermal conversion performances of ICG and WP5⊃ICG, an 808 nm NIR laser was delivered through a quartz cuvette containing an aqueous dispersion (3 ml) of the sample with different concentrations (0–200 μg/ml), and the light source was an external adjustable power (1 W/cm^2^) 808-nm semiconductor laser device (LR-MFJ-808/1W, Changchun Femtosecond Technology Co. Ltd., China). The temperature was monitored by a thermometer and recorded once every 30 s. The temperature signals also recorded at different time intervals (0–10 min) were analyzed with FL-IR tools systems.

## Results and Disscussion

### Host-Guest Interaction

**FIGURE 1:**
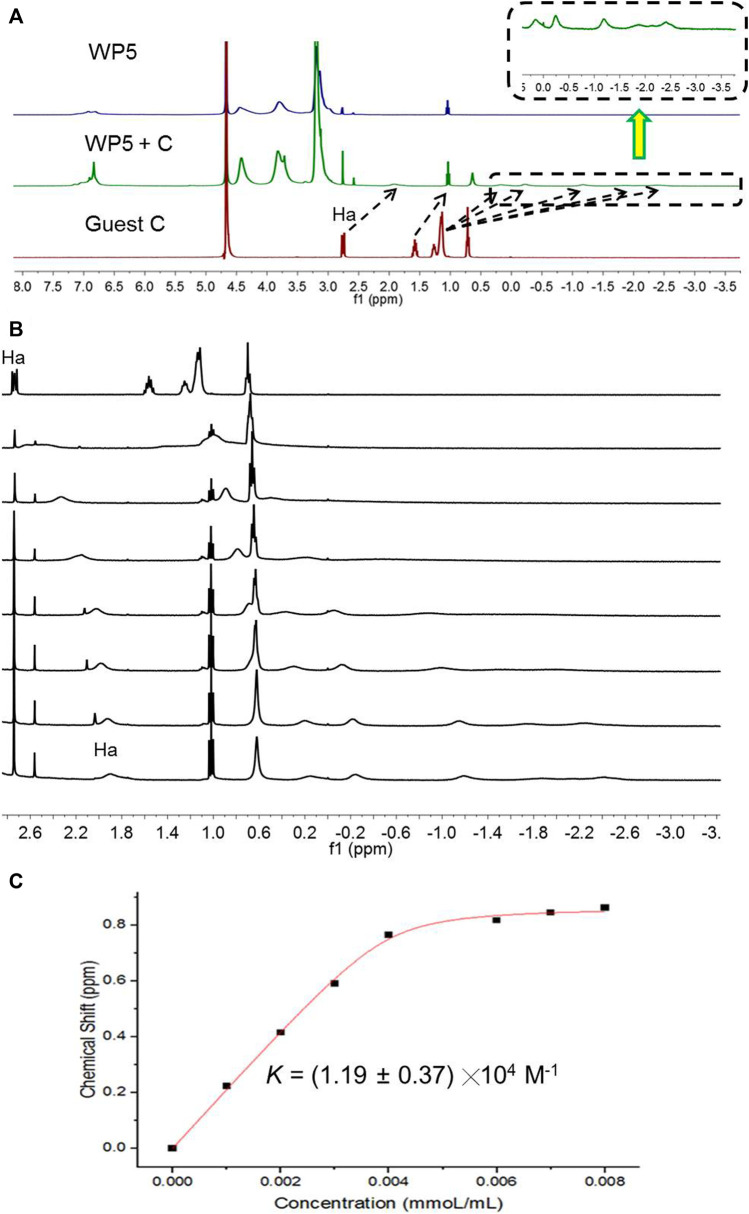
**(A)** ^1^H NMR spectra (D_2_O, 293 K, 400 MHz) of WP5 (16.00 mM), **(B)** WP5 (16.00 mM) + C (16.00 mM), and C (16.00 mM). **(C)** ^1^H NMR spectra (D_2_O, 293 K, 400 MHz) of C at a concentration of 16 mM with different concentrations (mM) of WP5. **(C)** The chemical shift changes of Ha on C upon addition of WP5.

As WP5 presents 10 quaternary ammonium cations on its macrocyclic framework, it can form a complex with anionic guests efficiently. The host-guest property of WP5 with anionic guests was investigated in detail, which revealed that WP5 displayed high affinities for sodium dodecyl sulfonate in aqueous solution. As shown in Figure 1, when WP5 and sodium dodecyl sulfate (**C**) were dissolved in water, the host-guest complex was formed. The ^1^H NMR spectra of an aqueous solution of WP5 (16.00 mM) and **C** (16.00 mM) showed that the complex was in fast exchange on the ^1^H NMR time scale and an upfield shift had taken place for Ha and Hb on guest **C** after complexation (Figure 1A). Furthermore, the overlapped signal corresponding to Hal was obviously split into five separate peaks (Figure 1A, right).

The above results showed that the linear guest **C** penetrated into the cavity of WP5 to form a [2]pseudorotaxane, the anion head of **C** was close to the trimethylammonium groups of WP5, and the Hal in the middle of alkyl chain lay in the cavity of pillar[5]arene, the H at the tail of **C** was outside the cavity (Figure 1A). The driving force for the formation of the complex was hydrophobic and electrostatic interactions. The hydrophobic cavity of WP5 could hold the hydrophobic alkyl chain of **C** and the cationic trimethylammonium groups of WP5 could bind the anionic sulfonate group of **C** via electrostatic interaction seen through 2D Nuclear Overhauser Effect Spectroscopy (NOESY). As shown in Supplementary Figure S6, the hydrogens of the alkyl chain on **C** were close to the pillar[5]arene platform because Hal showed a strong correlation with Hph, indicating that the alkyl chain was in close proximity to the cavity.

We then added the ^1^H NMR titration of WP5 into the aqueous solution of **C** (16.00 mM) to investigate the ability of the WP5 complex with C (Figure 1B). As shown in Figure 1B, the proton NMR signal related to Ha shifted upfield considerably with the increase of the concentration of WP5. When the mole ratio of WP5/**C** was 1, the Ha protons of **C** shifted upfield about 0.90 ppm while Hal protons on **C** shifted upfield even more. However, when the concentration of WP5 was higher than 16.00 mM, chemical shifts of Ha protons were almost unchanged, indicating the formation of a 1: 1 complex between WP5 and **C** in water. The association constant (*Ka*) of WP5⊃**C** was calculated to be (1.19 ± 0.37) ╳ 10^4^ M^−1^ by using a nonlinear curve-fitting analysis (Figure 1C).

### Stability and Photothermal Conversion Investigation

**FIGURE 2:**
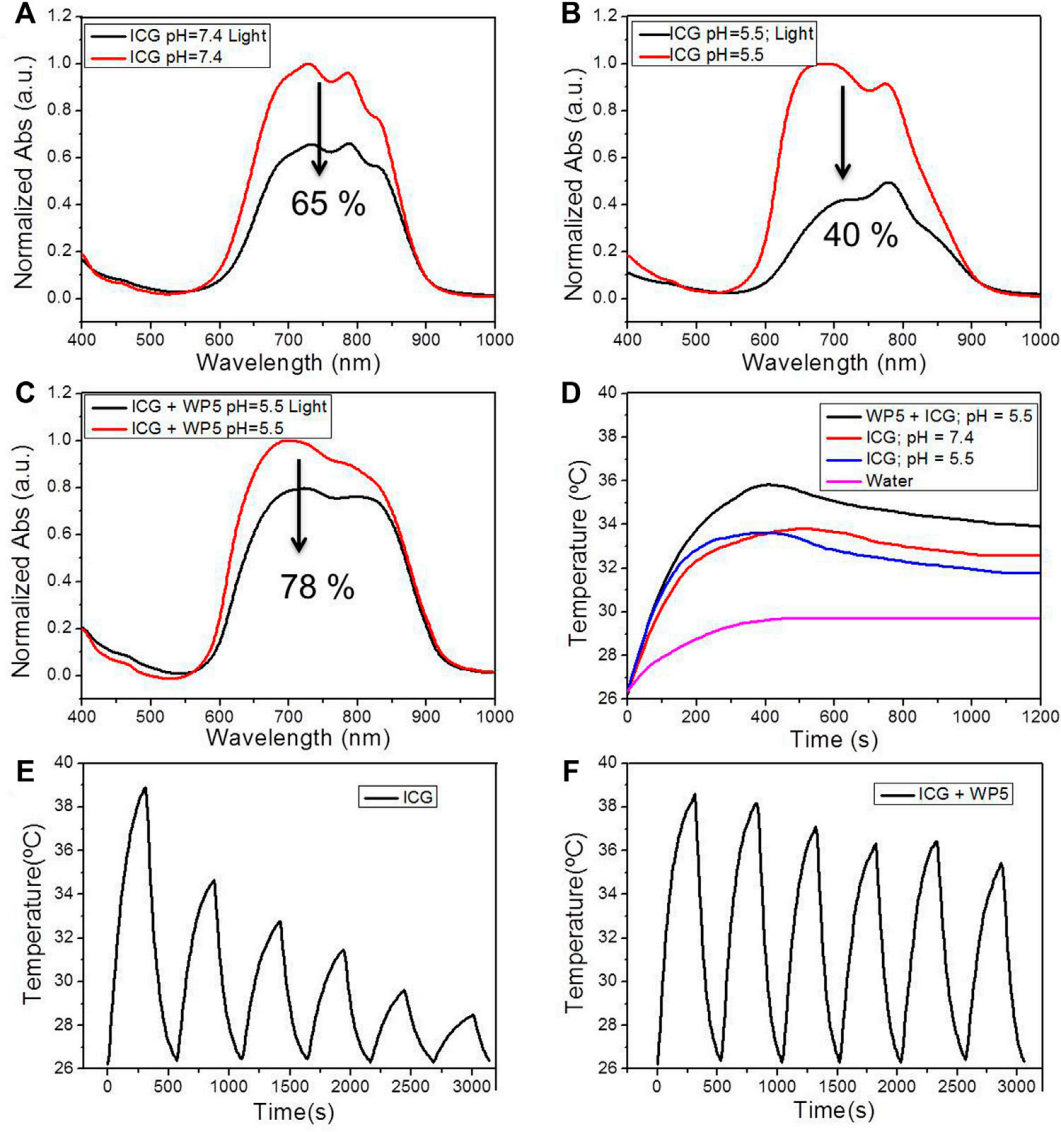
**(A)** UV-vis spectra of ICG (10 μg/ml) before and after irradiating with 808 nm light for 5 min when pH = 7.4. **(B)** UV-vis spectra of ICG (10 μg/ml) before and after irradiating with 808 nm light for 5 min when pH = 5.5. **(C)** UV-vis spectra of ICG (10 μg/ml) + WP5 before and after irradiating with 808 nm light for 5 min when pH = 5.5. **(D)** Photothermal conversion behavior of water, ICG (pH = 5.5), ICG (pH = 7.4), and WP5⊃ICG (pH = 5.5) under 808 nm laser irradiation (1.0 W cm^−2^). **(E)** Temperature variations of ICG (pH = 5.5) under 808 nm laser irradiation over six cycles of heating/cooling. **(F)** Temperature variations of WP5⊃ICG (pH = 5.5) under 808 nm laser irradiation over six cycles of heating/cooling.

After confirming the host-guest interaction between WP5 and **C**, we used WP5 to enhance the stability and photothermal conversion efficiency of ICG in water. From the UV-vis spectra, we found that ICG exhibited a strong absorption peak in the wavelength range of 600–900 nm (Figure 2A), which indicated that ICG could absorb near-infrared light well, and provided the possibility for photothermal therapy. Then we investigated the stability of ICG under illumination in water. After being irradiated with 808 nm light (1 W/cm^2^) for 30 min, the absorption of the pure ICG solution both in neutral (Figure 2A) or weak acidic (Figure 2B) environments was decreased significantly, indicating that ICG is unstable and easily degraded under illumination, especially in acid conditions. However, for the WP5⊃ICG group, the absorption peak of ICG decreased less under the same conditions, indicating that the host-guest complexation could protect ICG from degradation under light conditions (Figure 2C). We further studied the photothermal conversion efficiency of ICG in the aqueous solution. As shown in Figure 2D, with the increase of acid, the photothermal conversion efficiency of ICG decreased. But when forming the WP5⊃ICG complex, even at pH = 5.5, the temperature rose higher than that of pure ICG (Figure 2D). Infrared thermal images confirmed that the WP5⊃ICG complex could prove the photothermal conversion efficiency of ICG (Supplementary Figure S7). Importantly, by measuring the temperature change of ICG and WP5⊃ICG under the 808 nm laser and pH = 5.5 for six on/off laser cycles, we found that with each cycle, the increase in temperature dropped sharply for pure ICG (Figure 2E), but for WP5⊃ICG, it still increased by about 70% of the maximum temperature after six cycles (Figure 2F). These experimental results show that ICG is more easily degraded by light in weak acid conditions, and after forming a host-guest complex with WP5, WP5 can protect ICG and improve its photothermal conversion efficiency and stability.

### Cell Viability (MTT) Assay

**FIGURE 3:**
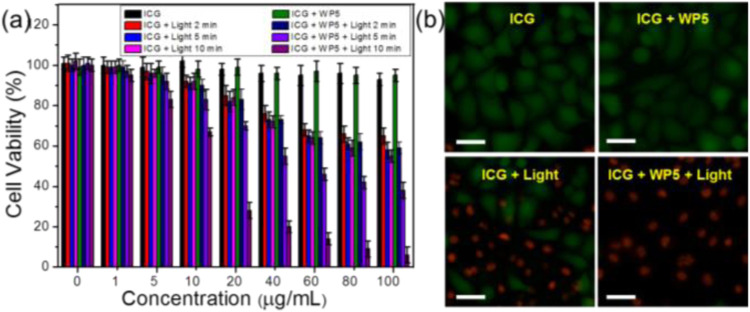
**(A)** Cell viabilities of HeLa cells treated with different groups with 808 nm laser irradiation (1 W/cm^2^). Error bars were based on the standard error of mean (*n* = 4). **(B)** Fluorescence images of calcein AM (live cells, green) and PI (dead cells, red) co-stained HeLa cells after different treatments (ICG = 100 μg/ml, irradiated for 10 min).

The above results suggest that WP5⊃ICG can be used as an excellent photothermo-therapeutic agent in the weak acidic microenvironment of tumor tissue. So we cultured HeLa cells with ICG and WP5⊃ICG at a certain concentration, and irradiated them with 808 nm of near-infrared light for different lengths of time. The relative activity of HeLa cells was measured by an MTT assay. The treated cells were double-stained with calcein AM/propidium iodide (PI) to differentiate the living cells from the dead cells. As shown in Figure 3A, ICG and WP5⊃ICG did not show cytotoxicity with increasing concentration in the absence of light, but both ICG and WP5⊃ICG showed cytotoxicity in the presence of near-infrared light. Moreover, the cytotoxicity of WP5⊃ICG was stronger than that of ICG. The cytotoxicity of ICG was found to be unchanged after 2 min of laser irradiation, indicating that ICG was degraded in tumor tissue after 2 min of laser irradiation, no further light conversed into heat to kill cancer cells. However, for WP5⊃ICG, the cytotoxicity continued to increase over time, suggesting that with the protection of WP5, ICG can continuously and steadily transform light into heat to kill cancer cells. The fluorescence imaging data of the cells were consistent with the relative cytotoxicity studies. As shown in Figure 3B, the green color represents living cells, and the red represents dead cells. We can clearly see that all the cancer cells died when treated with the WP5⊃ICG + light group while for ICG + light, only 60% of cancer cells died. In conclusion, the complex formed by WP5 and ICG cannot only improve the photothermal conversion efficiency of ICG, but also greatly improve its stability, which makes WP5⊃ICG a promising photothermal therapeutic agent for cancer.

## Conclusion

A trimethylammonium functionalized cationic water-soluble pillar[5]arene (WP5) was designed and synthesized. It was found that WP5 and linear guest **C** could form a stable host-guest complex by ^1^H NMR. As ICG has two alkyl sulfonates, the host-guest interaction between WP5 and ICG cannot only inhibit the π-π stacking between ICG molecules, but can also improve the photothermal conversion efficiency of ICG in water. Moreover, ICG can also be protected by WP5 to reduce its degradation rate under light conditions and improve its stability. Cell experiments showed that WP5⊃ICG has a better ability to kill cancer cells under near-infrared light than ICG in the weak acidic microenvironment of tumor tissue. Moreover, as WP5⊃ICG is more stable, it can kill cancer cells when exposed to light continuously. The method of improving the stability and conversion efficiency of photothermal reagents through the host-guest interaction provides a new idea for cancer therapy. Our following study will focus on animal experiments.

## Data Availability

The original contributions presented in the study are included in the article/Supplementary Material, further inquiries can be directed to the corresponding authors.
