# Supplementary material for: Enhancing the Stability and Photothermal Conversion Efficiency of ICG by Pillar[5]arene-Based Host-Guest Interaction
Source: Front Chem. 2021 Oct 29;9:775436. doi: 10.3389/fchem.2021.775436 (PMC8586498; doi:10.3389/fchem.2021.775436)
Supplement: Supplementary file 1 [file DataSheet1.doc]

**Supporting Information**

Enhancing the stability and photothermal conversion efficiency of ICG by pillar[5]arene-based host-guest interaction

Yue Ding,^§^ Chenwei Wang,^§^ Bing Lu*, and Yong Yao*

School of Chemistry and Chemical Engineer, Nantong University, Nantong, Jiangsu, 226019, P.R. China,

Correspondence: 2020028lubing@ntu.edu.cn; yaoyong1986@ntu.edu.cn

§Yue Ding and Chenwei Wang contribution equally to this article

^1^H NMR and ^13^C NMR spectra of compounds **A**, **B,** and **WP5**


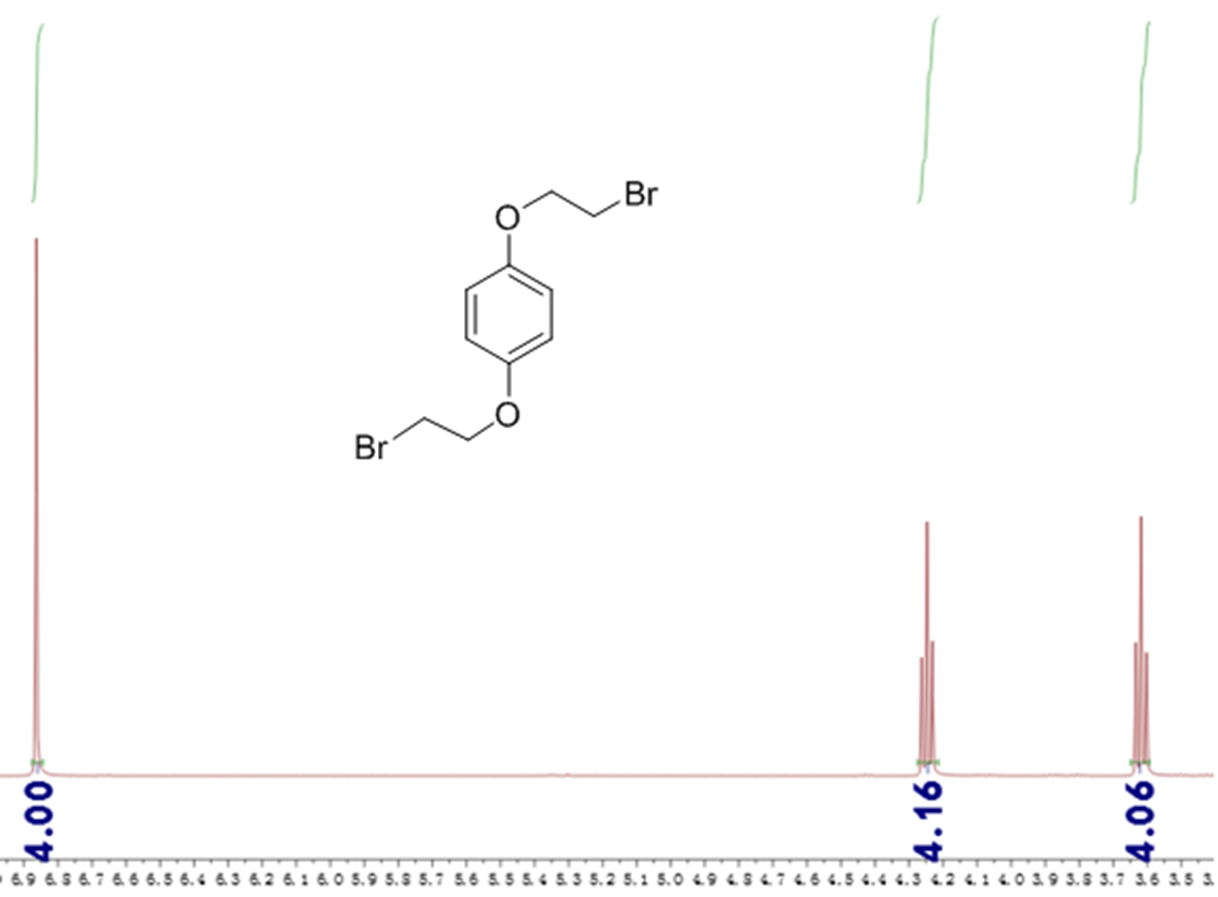


Figure S1. ^1^H NMR spectra of **A** in CDCl3 at 400 MHz.


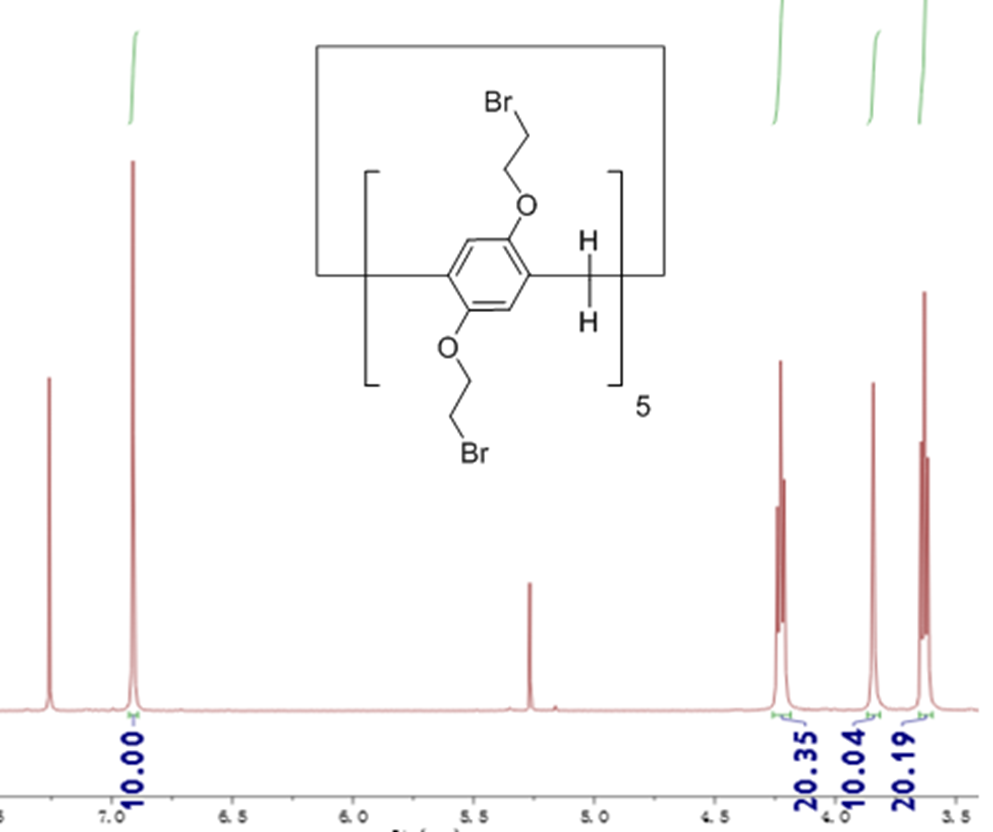


Figure S2. ^1^H NMR spectra of **B** in CDCl3 at 400 MHz.


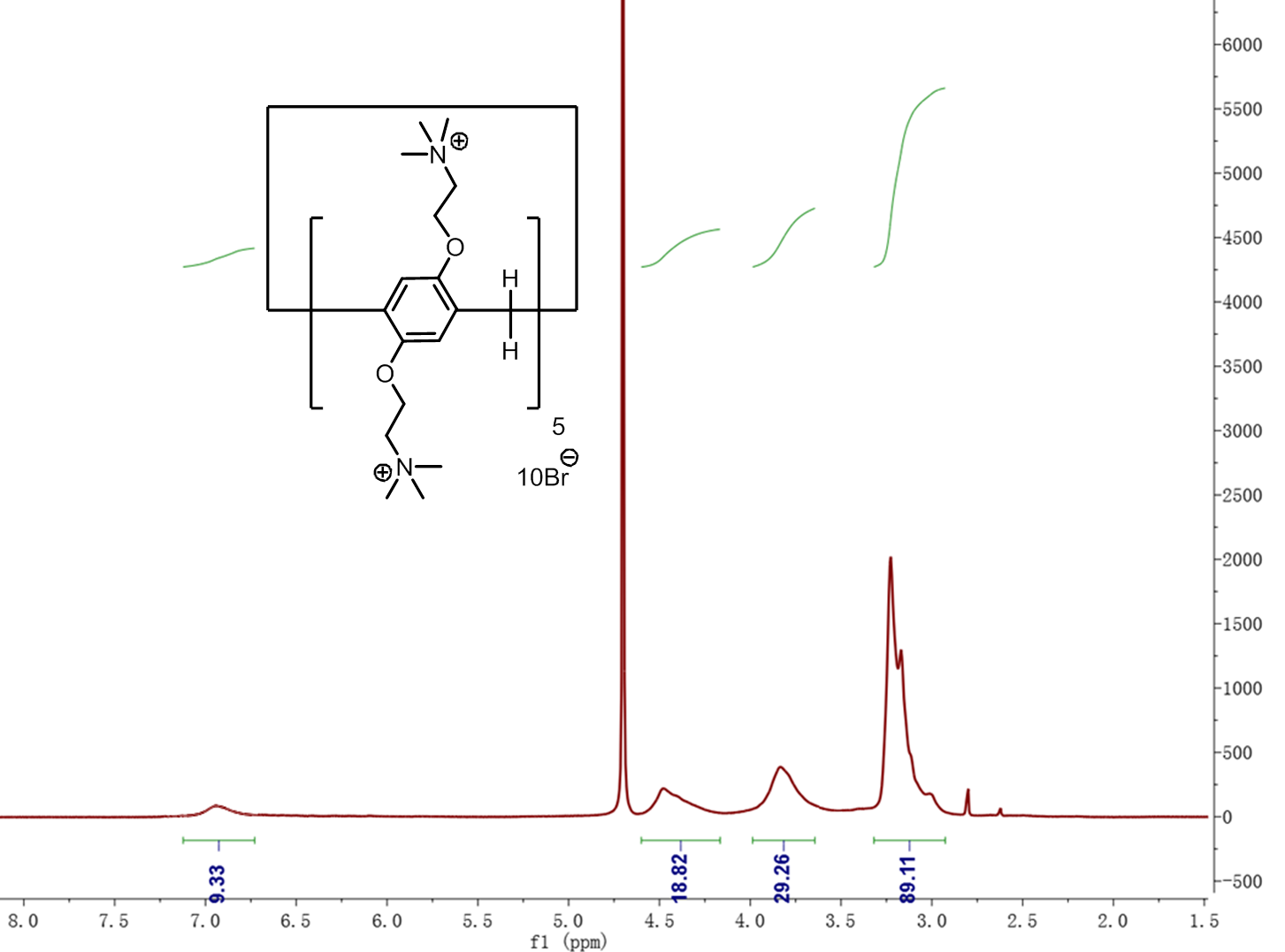


Figure S3. ^1^H NMR spectra of **WP5** in D_2_O at 400 MHz.


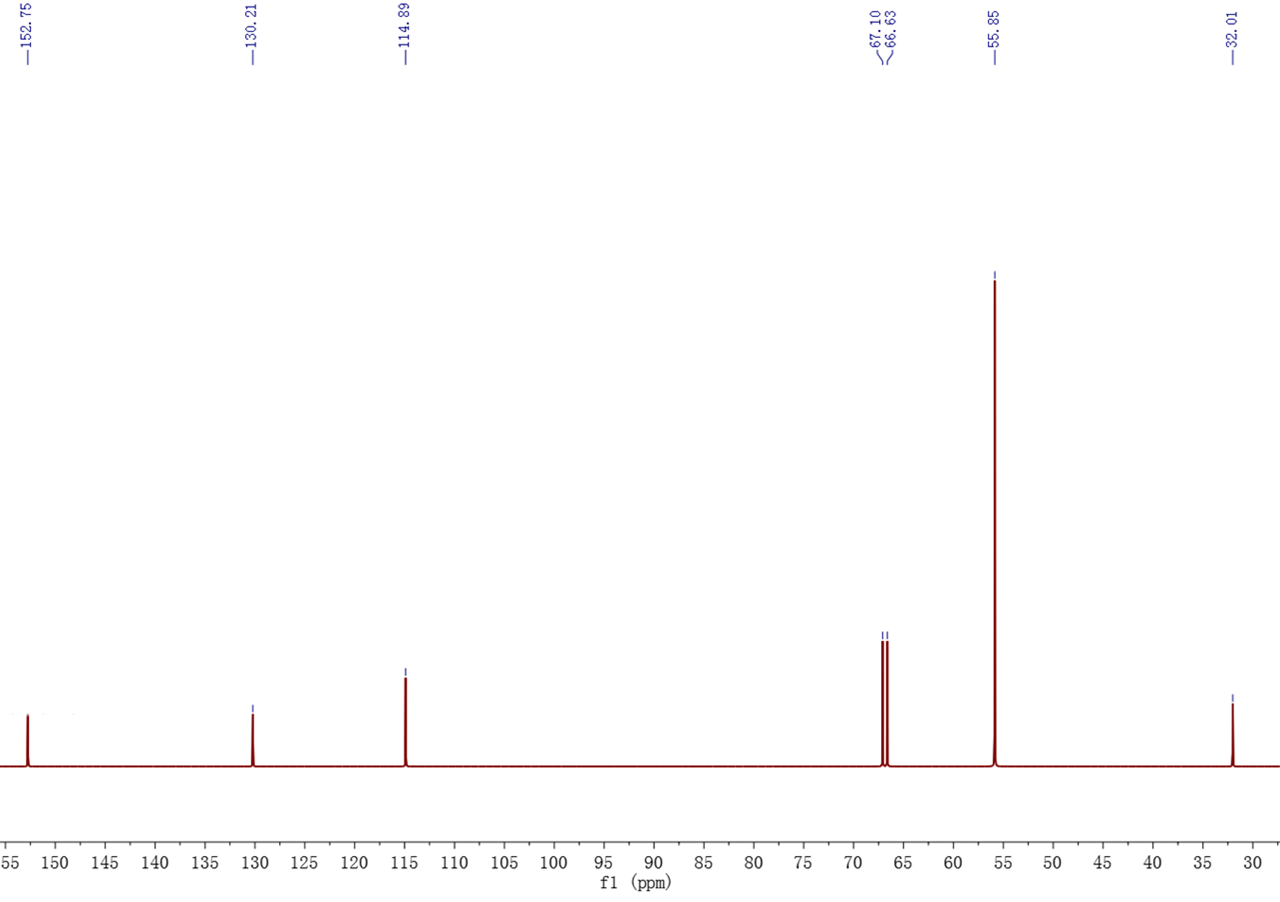


Figure S4. ^13^C NMR spectra of **WP5** in D_2_O at 100 MHz.


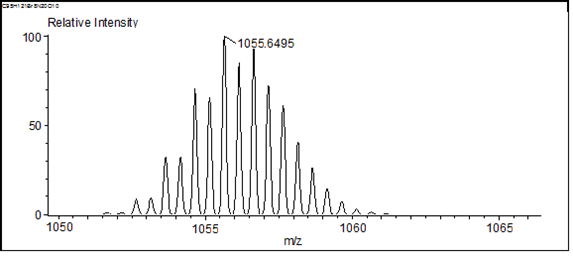


Figure S5. High resolution electrospray ionization mass spectra of **WP5** [M – 2 Br^-^]^2+^.


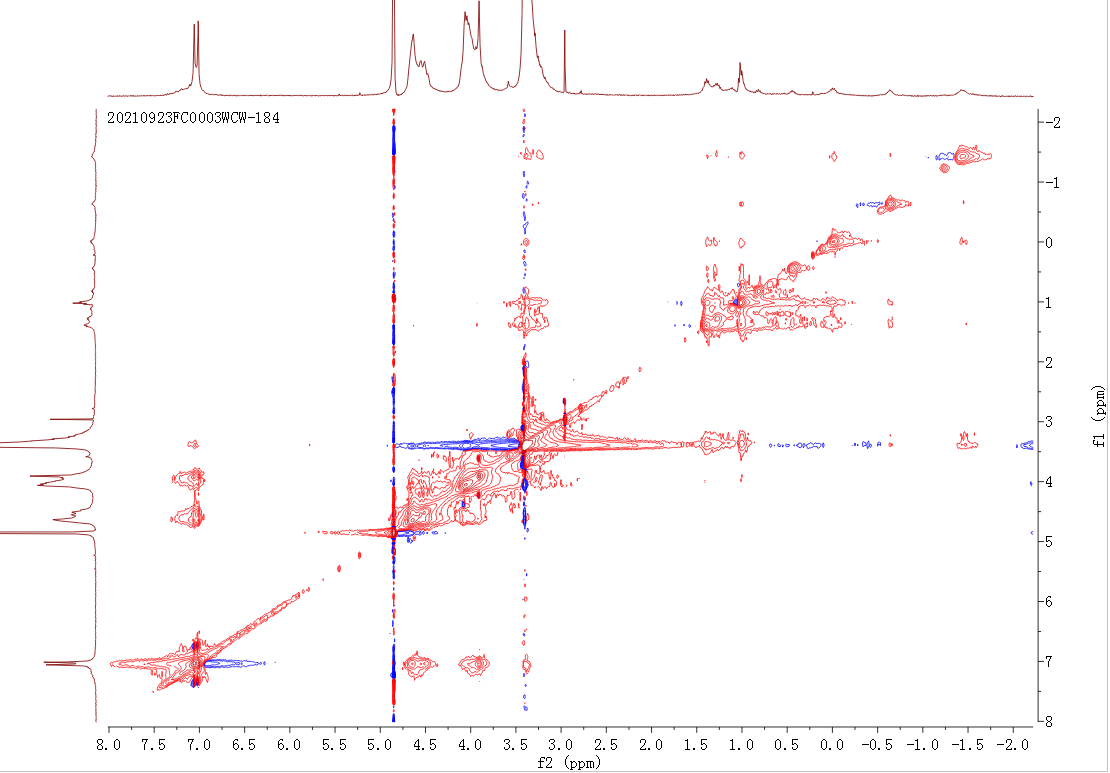


Figure S6. Partial 2D NOESY spectrum of a D_2_O solution of WP5⊃C at 298K.


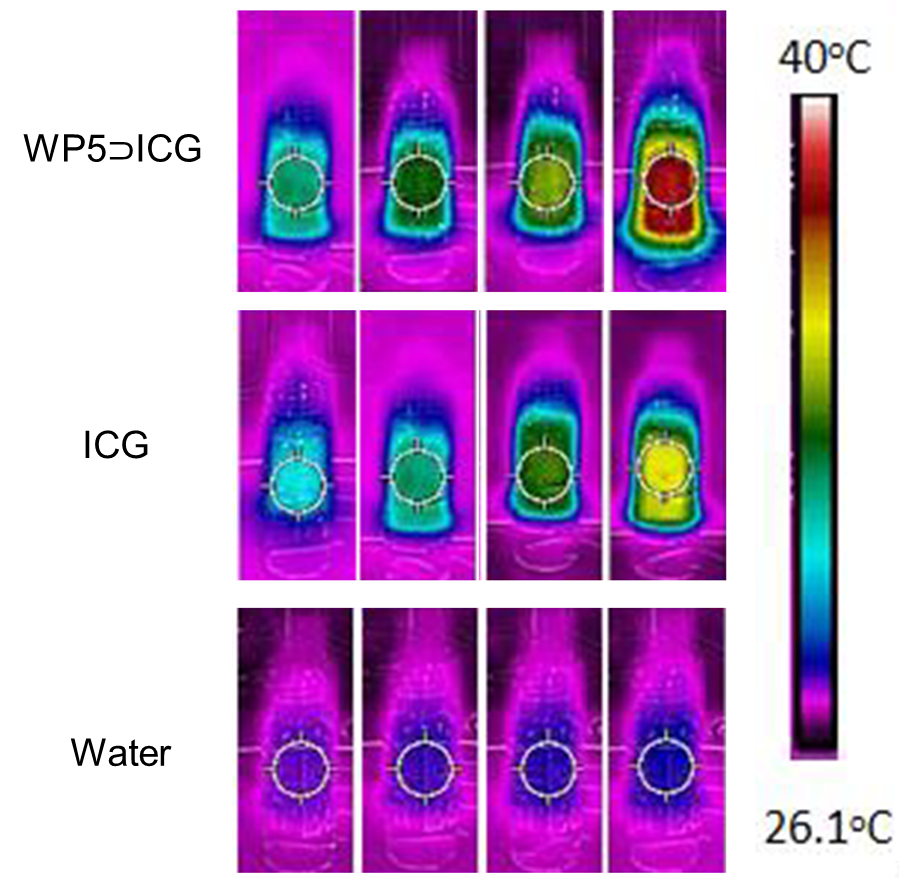


Figure S7. Infrared thermal images of WP5⊃ICG (10 μg/mL), ICG (10 μg/mL), and deionized water after the treatment with an 808 nm laser (1.0 W/cm^2^).
